# *In Vitro* Interactions between 17β-Estradiol and DNA Result in Formation of the Hormone-DNA Complexes

**DOI:** 10.3390/ijerph110807725

**Published:** 2014-07-31

**Authors:** Zbynek Heger, Roman Guran, Ondrej Zitka, Miroslava Beklova, Vojtech Adam, Rene Kizek

**Affiliations:** 1 Department of Ecology and Diseases of Game, Fish and Bees, Faculty of Veterinary Hygiene and Ecology, University of Veterinary and Pharmaceutical Sciences, Palackeho 1–3, CZ-612 42 Brno, Czech Republic; E-Mails: heger@mendelu.cz (Z.H.); zitkao@seznam.cz (O.Z.); beklovam@vfu.cz (M.B.); 2 Department of Chemistry and Biochemistry, Faculty of Agronomy, Mendel University in Brno, Zemedelska 1, CZ-613 00 Brno, Czech Republic; E-Mails: R.Guran@email.cz (R.G.); vojtech.adam@mendelu.cz (V.A.); 3 Central European Institute of Technology, Brno University of Technology, Technicka 3058/10, CZ-616 00 Brno, Czech Republic

**Keywords:** cancer, denaturation, endocrine disruptors, estrogens, nucleic acids, spectrometry

## Abstract

Beyond the role of 17β-estradiol (E_2_) in reproduction and during the menstrual cycle, it has been shown to modulate numerous physiological processes such as cell proliferation, apoptosis, inflammation and ion transport in many tissues. The pathways in which estrogens affect an organism have been partially described, although many questions still exist regarding estrogens’ interaction with biomacromolecules. Hence, the present study showed the interaction of four oligonucleotides (17, 20, 24 and/or 38-mer) with E_2_. The strength of these interactions was evaluated using optical methods, showing that the interaction is influenced by three major factors, namely: oligonucleotide length, E_2_ concentration and interaction time. In addition, the denaturation phenomenon of DNA revealed that the binding of E_2_ leads to destabilization of hydrogen bonds between the nitrogenous bases of DNA strands resulting in a decrease of their melting temperatures (*T*_m_). To obtain a more detailed insight into these interactions, MALDI-TOF mass spectrometry was employed. This study revealed that E_2_ with DNA forms non-covalent physical complexes, observed as the mass shifts for app. 270 Da (Mr of E_2_) to higher molecular masses. Taken together, our results indicate that E_2_ can affect biomacromolecules, as circulating oligonucleotides, which can trigger mutations, leading to various unwanted effects.

## 1. Introduction

Contaminants of emerging concern (CECs), such as endocrine disrupting compounds (EDCs), are organic contaminants that have been detected in wastewater, surface water, and drinking water throughout the world [1,2,3,4]. Among the EDCs, estrogen hormones have become emerging pollutants, because of their presence in various environmental compartments and concerns about their estrogenic effects to wildlife and humans [5,6,7]. Humans can be exposed to estrogens on a daily basis, especially because of their presence in drinking water [4]. The increased exposure of estrogen hormones, among which the largest potency is dedicated to 17β-estradiol (E_2_) [8,9,10], may cause abnormal reproduction, dysfunctions of neuronal and immune systems, or stimulation of cancer cells proliferation [11,12,13,14].

**Figure 1:**
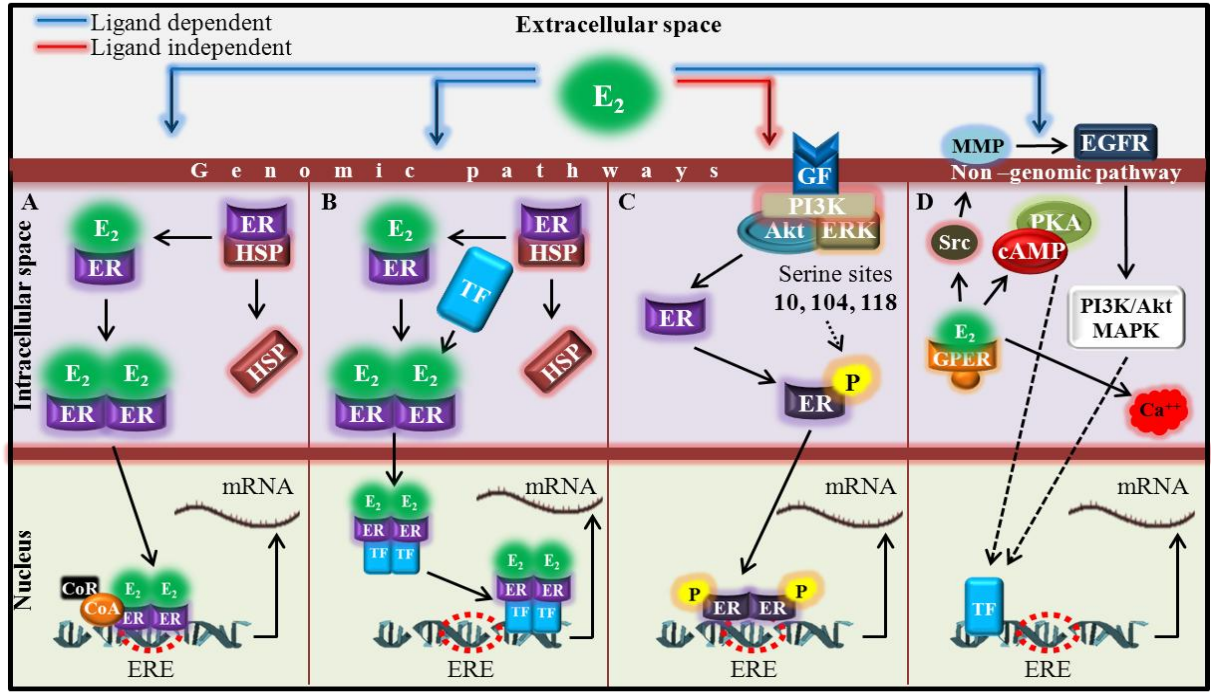
Model expressing the distinct molecular pathways of ER transcription factors regulatory actions. (**A**) The classical direct, genomic ligand-dependent pathway includes detachment of transcription factors—Estrogen receptors (ER) from complex with heat shock proteins (HSP) and shift of E_2_-ER complex towards estrogen response element (ERE), localized in nucleus, where transcription is triggered after recruitment of coactivators (CoA) and corepressors (CoR) of transcription process [24]. (**B**) Non-direct genomic ligand-dependent mechanism—Tethering or cross-talk includes protein-protein interaction with other transcription factors (TF) after ligand activation, and thereby gene regulation is affected by indirect DNA binding [29]. (**C**) Estrogen receptors can be phosphorylated at specific serine sites by growth factors (GF) and other plasma membrane estrogen receptors, coupled to kinase signaling (ERK-extracellular regulated kinase, Akt—Protein kinase B, PI3K—Phosphoinositide 3-kinase). Phosphorylated (P) ER can trigger the transcription in a ligand-independent manner [26]. (**D**) The non-genomic effect includes the activation of receptor, associated with the membrane receptors including G-protein-coupled receptor (GPER). Signaling through GPER occurs via transactivation of the epidermal growth factor receptor (EGFR) activated by metalloproteinases (MMP). This phenomenon leads to downstream activation of various signaling molecules, triggering possible alterations in protein synthesis. Furthermore, cyclic adenosine monophosphate (cAMP) and intracellular calcium mobilization (Ca^++^) are stimulated by E_2_-mediated activation of GPER. This leads to rapid physiological responses without gene regulation. Moreover, GPER also regulates transcriptional activity by other signaling mechanisms as cAMP, ERK or PI3K [27].

Currently, more than 70% of breast carcinomas are found to be estrogen receptor positive (ER+) [15], exhibiting the positive proliferative effects as a response to the presence of estrogens [16,17]. Although estrogen exposure is now a widely accepted risk factor in breast cancer development, the mechanisms through which estrogens induce breast carcinogenesis have not been described satisfactorily. One of the generally accepted mechanisms includes metabolism of E_2_ via cytochrome P450-mediated oxidation of catechol estrogens to quinones that react with DNA and form estrogen-DNA adducts. The resulting critical mutations can trigger the development of breast and also other human malignancies [18,19]. Estrogens may further affect cell proliferation via several genomic and non-genomic pathways [20,21]. The mechanisms of genomic signaling of estrogens have been reported by several researchers [22,23,24,25,26] (shown in Figure 1A,B), whereas the non-genomic mechanisms are still not well understood (shown in Figure 1C,D). The most likely hypothesis seems to be the activation of signaling cascades via second messenger as cAMP [27], which subsequently initiates the action of protein kinase A, or mobilize the intracellular calcium in a rapid manner [28]. Both, models representing genomic and non-genomic signaling pathways are shown in Figure 1A–D.

Whereas several actions of E_2_ are caused by its reactive metabolites, there exist also possible effects, resulting from excessive exposure to unmetabolized E_2_ *per se*. Hence, the aim of this study is to identify the possible interactions between 17β-estradiol, the most potent natural estrogen, and double-stranded DNA, the key macromolecule reacting with carcinogens. We suggested four dsDNA fragments imitating short sections of the natural DNA sequences comprising estrogen response elements (ERE) as the promoter regions for estrogen-driven transcription [29]. DNA fragments are found to be varied in their length and nitrogenous bases composition. After interactions with E_2_, their behavior was observed using UV-vis spectrophotometry and matrix-assisted laser desorption/ionization time of flight (MALDI-TOF) mass spectrometry in a timely manner.

## 2. Experimental Section

### 2.1. Chemicals and pH Measurement

Working solutions as buffers or standard solution of 17β-estradiol were prepared daily by diluting the stock solutions. The interactions of E_2_ and DNA fragments were carried out in the environment maintained by phosphate buffered saline (PBS, pH = 7.4), whose osmolarity and ion concentrations match with those of the isotonic environment in human body. PBS was prepared according to the protocol as follows: 8.01 g∙L^−1^ of NaCl, 0.20 g∙L^−1^ of KCl; 1.78 g∙L^−1^ of Na_2_HPO_4_·H_2_O and 0.27 g∙L^−1^ of KH_2_PO_4_, dissolved MilliQ water, obtained by reverse osmosis using Aqual 25 (Aqual s.r.o., Brno, Czech Republic). The water was purified by apparatus Direct-Q 3 UV Water Purification System equipped with the UV lamp, purchased from Millipore (Billerica, MA, USA) with resistance established to 18 MΩ·cm^−1^. 17β-estradiol, oligonucleotides and others were purchased from Sigma Aldrich (St. Louis, MO, USA) in ACS purity, unless noted otherwise. The pH was measured using pH meter WTW inoLab (Weilheim, Germany).

### 2.2. Oligonucleotides

Sequences used for experiments were designed as it is shown in Table 1.

**Table 1:** Oligonucleotide sequences employed for interaction experiments.

ERE_1_	5’-CTAATCACTCTGACCAT-3’ 3’-GATTAGTGAGACTGGTA-5’
ERE_2_	5’-CCAGGTCAGAGTGACCTGAG-3’ 3’-GGTCCAGTCTCACTGGACTC-5’
ERE_3_	5’-GCAGGTCAGAGTGACCTGAGCTAG-3’ 3’-CGTCCAGTCTCACTGGACTCGATC-5’
ERE_4_	5’-CCAGGTCAGAGTGACCTGAGCTACGGTGACACAGGCAG-3’ 3’-GGTCCAGTCTCACTGGACTCGATGCCACTGTGTCCGTC-5’

### 2.3. The Hybridization Process of Single-Stranded DNA Fragments

The double-stranded DNA fragments were acquired by hybridization of two complementary single-stranded DNA oligonucleotides in ratio 1:1, with volume of 30 µL. Subsequently, 30 µL of immobilization solution (composed of 100 mM Na_2_HPO_4_ + 100 mM NaH_2_PO_4_), 0.5 M NaCl, 0.6 M guanidinium thiocyanate, 0.15 M Trizma base adjusted by HCl on pH of 7.5 was added to the solution containing oligonucleotides. The resulting solution was stirred for 40 min to obtain the final dsDNA fragments.

### 2.4. Spectrophotometric Analysis of Interactions between E_2_ and DNA Fragments

Two hundred µL of mixture, comprising E_2_ (final concentrations in range 0, 0.625. 1.25, 2.5, 5, 10, 20 and 40 nM) and oligonucleotides representing EREs (final concentration of 200 nM) was analyzed using UV-vis spectrophotometer SPECORD 210 (Analytik Jena, Jena, Germany). Prior to each measurement, the resulting complex was filtered through Amicon 3k (Millipore, Billerica, MA, USA). Carousel was heated to 37 °C by a flow thermostat Julabo F25 (Julabo, Seelbach, Germany) and the absorption characteristics of DNA representing EREs (A_λmax_ = 260 nm) were determined during 0–120 min lasting of interaction. For the subsequent denaturation study, the same final concentrations of E_2_ (0–40 nM) and DNA (200 nM) were employed. The denaturation study was carried out within the temperature ranges from 25 to 80 °C per 1.5 °C every 90 s using UV-vis spectrophotometer SPECORD S600 (Analytik Jena, Jena, Germany) by measuring of absorption maxima of DNA as A_λmax_ = 260 nm. Denaturation analyses were carried out with E_2_/DNA mixture after 120 min of lasting interaction.

### 2.5. MALDI-TOF MS Analysis of E_2_/DNA Fragments Interaction

Interaction of each ERE oligonucleotide in final concentration of 50 nM with E_2_ in final concentration of 20 nM was analyzed using matrix assisted laser desorption/ionization-time of flight (MALDI-TOF) mass spectrometer Bruker Ultraflextreme (Bruker Daltonik GmbH, Germany) equipped with a laser at the wavelength of 355 nm with an accelerating voltage of 25 kV, cooled with nitrogen. Prior each measurement, the resulting complex was filtered through Amicon 3k (Millipore, Billerica, MA, USA). The matrix used for the analyses was 3-hydroxypicolinic acid (3-HPA) with addition of ammonium citrate (0.8 mg in 100 µL of water). The solutions for analyses were mixed in ratio of 1:1 (matrix/substance). After obtaining a homogeneous solution, 1 µL was applied on the target and dried under atmospheric pressure and ambient temperature (25 °C). The spectra were typically acquired from the average of 20 sub spectra from a total of 500 shots of the laser.

### 2.6. Descriptive Statistics

Mathematical analysis of the data and their graphical interpretation were realized by Microsoft Excel^®^, Microsoft Word^®^ and Microsoft PowerPoint^®^ (Microsoft, Redmond, WA, USA). Results are expressed as mean ± standard deviation (S.D.) unless noted otherwise.

## 3. Results and Discussion

On the basis of cellular functions, DNA is the key macromolecule, which is highly protected against damages coming from various substances including those called carcinogens [30,31]. Metabolism of estrogens via catechol estrogen pathway was previously characterized as a balanced set of activating and protective enzymes. The disruption of this homeostasis with excessive production of catechol estrogen quinones leads to interactions with DNA molecules resulting in inducement of mutagenesis and subsequent initiation of breast cancer [32]. Due to the fact that the metabolic conversion of E_2_ plays a crucial role in its adverse effects on organisms, there may exist other ways how the estrogen hormones (among which E_2_ exhibits the largest potency [10]) affect macromolecules as the nucleic acids. Therefore, for a better understanding of the fate of DNA upon contact with E_2_, we decided to perform an *in vitro* interaction study using spectrophotometry and mass spectrometry.

### 3.1. An Optical Behavior of DNA Affected by 17β-Estradiol

**Figure 2:**
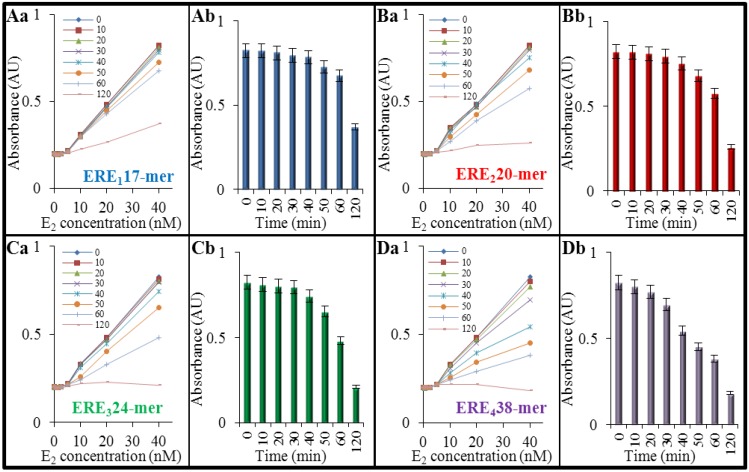
The expression of the interaction strength between DNA fragment and E_2_ obtained using UV-vis spectrophotometry. Various interaction times were tested (0–120 min), and influence of E_2_ (final concentrations used as 0, 0.625, 1.25, 2.5, 5, 10, 20 and 40 nM) on DNA fragment (200 nM) absorbance was determined at DNA absorption maxima (A_λmax_ = 260 nm). (**A**) ERE_1_, composed of 17 bp; (**B**) ERE_2_—20 bp; (**C**) ERE_3_—24 bp; (**D**) ERE_4_—38 bp. The shown absorbance values of 40 nM E_2_ in entire interaction time period (0–120 h) were marked with lowercase. (**Ab**) ERE_1_, (**Bb**) ERE_2_, (**Cb**) ERE_3_ and (**Db**) ERE_4_. The values are means of three independent replicates (*n* = 3).

Firstly, UV-vis spectrophotometry was employed to study 17β-estradiol interactions with DNA on the level of basic *in vitro* characteristics. To determine the interaction strength, the changes of DNA absorbance (final concentration 200 nM) at A_λmax_ = 260 nm after interactions with E_2_ (final concentrations in range 0–40 nM) were evaluated. As it is obvious from curves in Figure 2Aa–Da, shorter oligonucleotides were determined to exhibit weaker interaction in time, but 120 min were shown to be sufficient to change the optical attributes of all tested sequences of DNA. Subsequently, to better understand the obtained results, the DNA absorbance was converted into bar graphs. In these figures, the largest applied concentrations of E_2_ (40 nM) were presented for whole interaction interval. The shortest chain (17-mer) was shown to interact weakly. The most significant change in optical behavior was identified after 120 min (0.37 AU compared with absorbance of control—0 nM E_2_—0.82 AU) as it is shown in Figure 2Ab. In the case of longer sequences, more apparent decrease of their absorbance occurs after 120 min, particularly to the final 0.26 AU in DNA composed of 20 bp (Figure 2Bb), to 0.21 AU in DNA composed of 24 bp (Figure 2Cb) and 0.18 AU in the longest DNA composed of 38 bp (Figure 2Db).

It can be concluded, based on the obtained results, that within the time interval of 0–120 min, the interactions are fundamentally dependent on the three factors (I) oligonucleotide length; (II) time of interaction and (III) concentration of E_2_. Particularly, the longer DNA sequences provide more accessible binding sites for hormone interaction, which then results in formation of the complex under lower threshold concentrations and moreover, a shorter time is required for its establishment. Because nucleic acids absorb strongly in the UV region (260 nm) [33], the hypochromic effect under the influence of E_2_ is caused by changing of optical behavior of the complex, but not by disintegration of double-stranded structure that commonly leads to the increase of absorbance by unstacking of nitrogenous bases elevating absorbance of single-stranded DNA [34].

### 3.2. An Effect of 17β-Estradiol on Denaturation of Double-Stranded DNA

**Figure 3:**
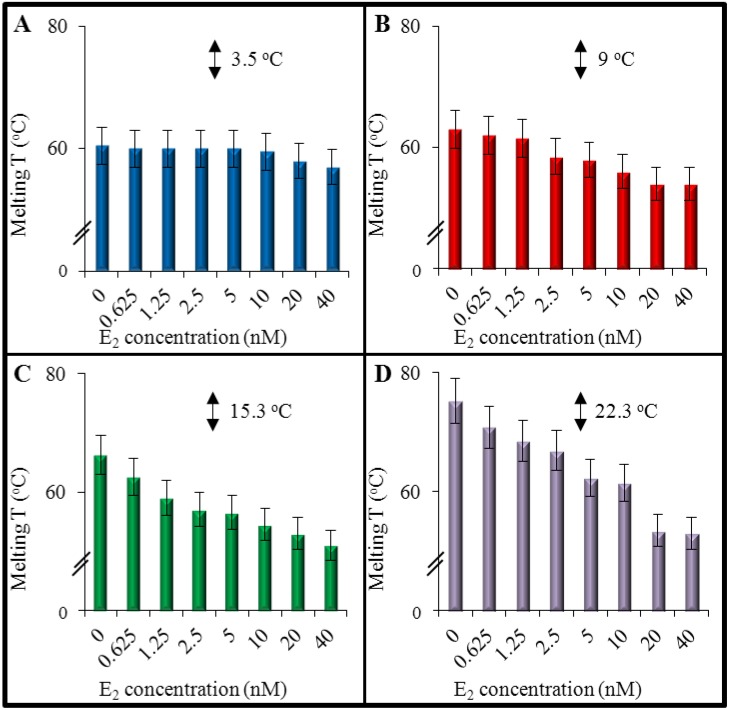
Melting temperatures (range 25–80 °C) of DNA (final concentration 200 nM) were evaluated after interaction with 17β-estradiol in final concentrations in range 0, 0.625, 1.25, 2.5, 5, 10, 20 and 40 nM and denaturation phenomenon is shown for: (**A**) ERE_1_; (**B**) for ERE_2_; (**C**) for ERE_3_; and (**D**) for ERE_4_ (interaction time—120 min, temperature increase step of 1.5 °C per 90 s). Absorbance was determined in A_λmax_ of DNA = 260 nm. The values are means of three independent replicates (*n* = 3).

Differences in energy between stacked G·C and A·T Watson-Crick base pairs in DNA duplex lead to complex melting profiles, observable with optical methods, providing a high degree of precision for the evaluation of thermodynamic quantities associated with the DNA stability [35]. Therefore, we decided to determine this DNA denaturation phenomenon using UV-vis spectrophotometry. In accordance with previous spectrophotometric results, we confirmed that the length of nucleic acid is closely related to willingness of the complexes formation. As it is shown in Figure 3A, melting temperature (*T*_m_) of the shortest DNA sequence (ERE_1_) exhibited *T*_m_ disparity at about 3.5 °C (subtraction of DNA +40 nM E_2_ from DNA with no E_2_ application after 120 min of lasting interaction). Despite the fact that in preliminary experiments, it was determined that 120 min of interaction are sufficient to form complex, and it was shown that the formation of the complex effect on the structure of ERE_1_ with 17-mer is not seen. This phenomenon points to a higher stability of shorter sequences against E_2_ and is closely related to sequence, where A·T pair is held by only two hydrogen bonds, while G·C has three, as it was described previously [36]. With increasing chain length, larger disparities between control and the highest applied E_2_ concentration (40 nM) were observed. As it can be seen in Figure 3B, in 20-mer ERE_2_ was observed 9 °C difference in *T*_m_. In case of 24-mer ERE_3_ the difference in *T*_m_ reached 15.3 °C (Figure 3C) and finally in 38-mer ERE_4_ 22.3 °C difference was determined (Figure 3D). Such a big melting abnormality is caused by a physical complexation of hormone with DNA, which leads to destabilization of the hydrogen bonds between the nitrogenous bases of DNA strands. This phenomenon further causes decrease of *T*_m_, in which the differential stability of A·T and G·C base pairs can be altered with a minimum of disturbance of the other properties of the native DNA helix [37].

### 3.3.MALDI-TOF Mass Spectrometry for Evaluation of E_2_-DNA Interaction

**Figure 4:**
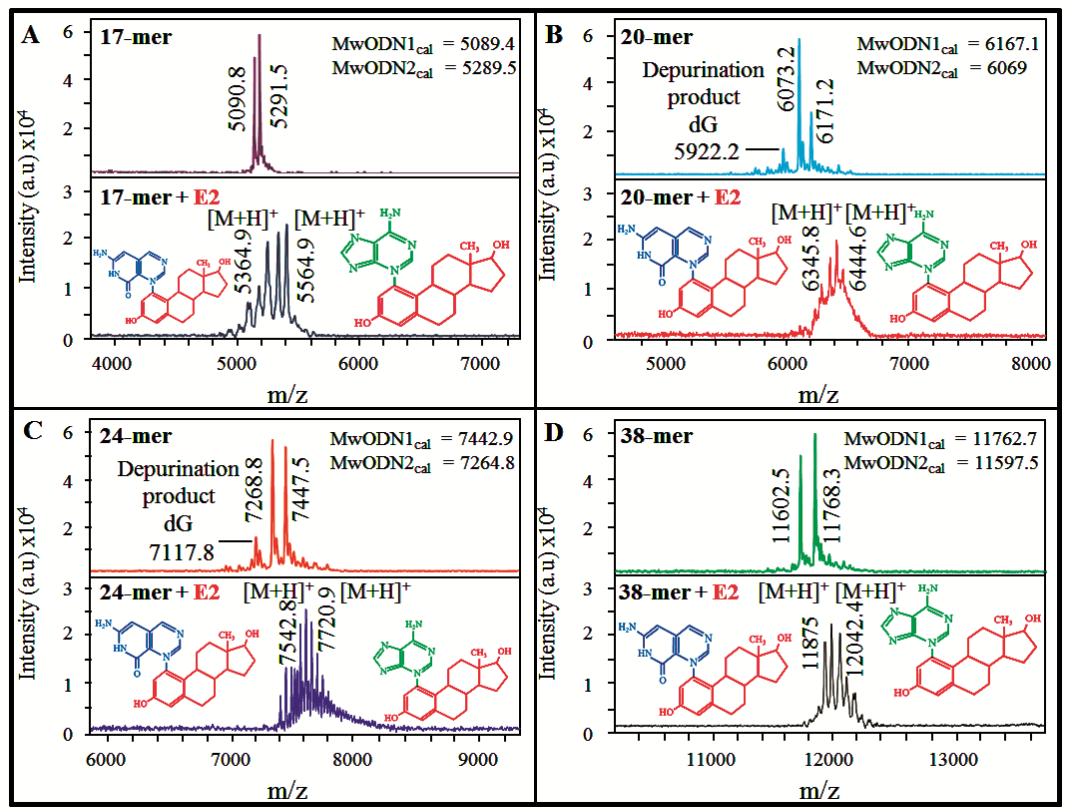
The matrix-assisted laser desorption/ionization time of flight (MALDI-TOF) mass spectra show the way of interaction between E_2_ (final concentration 20 nM) and each of DNA fragments (final concentration 50 nM) after 120 min. Spectra were carried out in linear, positive mode, using 3-hydroxypicolinic acid (3-HPA) as the matrix and laser output of 70%. The spectra show complex formation with (**A**) ERE1; (**B**) ERE_2_; (**C**) ERE_3_ and (**D**) ERE_4_. Upper spectra always show both—Calculated (MwODN_cal_) and experimentally determined molecular weight of DNA fragments. The lower spectra show the formation of complexes upon interaction with 20 nM E_2_.

To confirm the DNA/hormone complex formation, the MALDI-TOF analyses were carried out (Figure 4A–D). 3-hydroxypicolinic acid (3-HPA) which is commonly used as a DNA specific matrix was employed in this experiment [38,39,40]. Due to the presence of the negatively charged phosphate backbones, the adduct formation with alkali metal ions (Na^+^ or K^+^) is often observed in spectra of DNA. Hence a matrix additive represented by ammonium citrate was used to suppress undesirable cationization, whereas ammonium ions exchange with the Na^+^ and K^+^ ions, which are complexed with citrate [41]. The results of control analyses point to denaturation of double-stranded DNA. This phenomenon is caused by 3-HPA matrix as it was described previously, and results from protonation of bases that destabilize hydrogen bonds between two strands that partially denature a duplex upon mixing with 3-HPA [42]. Moreover, in the case of ERE_2_ and ERE_3_ control measurements, depurinating products, occurred as a result of heating (ionization with laser) and formed typical secondary peaks having 151 mass units (in the case of dG) less than major peak [43], which it is shown in Figure 4B,C. It was also shown that alkali metal adducts are completely absent in DNA spectra; thus, the ammonium citrate co-matrix performs well to eliminate them.

Subsequently, the interactions of DNA with 20 nM E_2_ were carried out and lasted 120 min. As it is shown in lower spectra in Figure 4A–D, all oligonucleotides formed complexes shifting by app. 270 Da (Mr of E_2_) to higher molecular masses. Moreover, the reduction of signal intensity and mass resolution occurred while increased the spectra complexity. This phenomenon is typical for oligonucleotides when forming the complexes [44]. Although both the purine and pyrimidine bases make up the structure of DNA contain nucleophilic sites suitable for reaction with carcinogens, the purines (adenine and guanine) are usually the most reactive sites for reaction [45]. Nevertheless, the metabolic conversion to reactive catechol estrogen quinones is fundamental for covalent interaction by Michael addition that results in depurinating adducts [18,46].

In organisms, the loss of these depurinating adducts generates apurinic sites and results in mutations in the *H-ras* oncogene by error-prone repair and subsequent mutations in H-ras protein that directs cells to grow and divide without control [47]. Moreover, the formation of covalent adducts with genotoxic carcinogens does not happen with DNA only, but also with all cellular nucleophilic species, e.g., protein, glutathione and/or water [48], but with not so profound genetic consequences as that on DNA.

Our *in vitro* study shows that excessive amount of E_2_ exhibits a potential to form the complexes with nucleic acids, and thus affect their attributes, which result in various pathological phenomena, with no need of conversion via cytochrome P450 pathway to more reactive products. Due to the fact that E_2_ is not chemically reactive, the strong complexes arise as a consequence of non-covalent physical reaction as it has been previously demonstrated by Saeed and coworkers with synthetic estrogen diethylstilbestrol (DES) [49]. Hendry and colleagues employed computational approach and they demonstrated that in partially unwound DNA there can be accommodated a variety of small molecules [50]. Estradiol was shown to be hypothetically inserted between base pair in DNA, using its hydroxyl groups at 3- and 17β-positions to form hydrogen bonds to phosphate oxygens on adjacent strands of DNA, because E_2_ contains heteroatoms separated by internuclear distances similar to that of phosphate oxygens on strands of dsDNA [51]. Based on our results, it can be stated that estradiol fits well within the topography of the double-helix molecule of DNA and physical complexes that can potentially affect the physiological behavior of the DNA and may form essential precursors for covalent bonds being formed between DNA and reactive estrogen metabolites.

Because of steroidal hormone properties, estrogens may pass through the phospholipid membranes of the cell to accomplish the interactions with circulating nucleotides as miRNA and/or siRNA to disturb their physiological functions. The RNA is not complexed with histones or embedded in chromatin structure, hence nucleotides are particularly sensitive to the binding of genotoxic agents of environmental origin [52]. It is conceivable that environmental carcinogens may modify the structure of miRNAs, thus blocking their access to the catalytic pockets of Dicers and arresting the miRNA maturation process. However, in the case of E_2_ complexes with these molecules, the real biological significance has to be elucidated through further, comprehensive *in vivo* experiments.

## 4. Conclusions

In our study, we showed that increased levels of hormone 17β-estradiol (E_2_) exhibit the potential to form complexes directly with double-stranded DNA. Using spectrophotometry and mass spectrometry it was shown that three major factors crucially influence the interaction: (I) the complexes were observed earlier if higher E_2_ concentration was used; (II) on the other hand increased time of interaction was shown to require lower concentrations of hormone to form complexes; and (III) it was revealed that longer oligonucleotide sequences form complex more readily probably due to higher content of E_2_ binding sites in their sequences. The stability of longer oligonucleotides was shown to be more influenced by hormone, probably due to a higher amount of binding sites causing destabilization of hydrogen bonds in DNA strands. Finally, the formation of the complexes was revealed by mass spectrometry MALDI-TOF, where shifts by about 272.38 Da were observed, pointing to the ability of E_2_ to bind the DNA and form the strong complexes as a result of non-covalent physical interaction, changing the optical and spectrometrical attributes of DNA.
